# Believing in Karma: The Effect of Mortality Salience on Excessive Consumption

**DOI:** 10.3389/fpsyg.2019.01519

**Published:** 2019-07-08

**Authors:** Siyun Chen, Haiying Wei, Lu Meng, Yaxuan Ran

**Affiliations:** ^1^School of Management, Jinan University, Guangzhou, China; ^2^School of Business, Renmin University of China, Beijing, China; ^3^School of Business Administration, Zhongnan University of Economics and Law, Wuhan, China

**Keywords:** belief in karma, mortality salience, terror management, temporal perspective, excessive consumption

## Abstract

This research proposes that mortality salience leads individuals to engage in differentiation of excessive consumption based on their appraisal of the karmic system. Study 1 demonstrated that mortality salience interacts with belief in karma to jointly determine excessive consumption, such that consumers faced with mortality salience tend to increase overconsumption likelihood when they have a weak (vs. strong) belief in karma. Study 2 revealed the underlying mechanism – temporal perspective – that drives our main effect. Replicating the findings of the two previous studies, study 3 further delineated benefit appeal as a theoretically derived boundary condition for the proposed interaction effect on excessiveness. Theoretical and, practical implications, as well as avenues for future research are discussed.

## Introduction

Mortality cues are ubiquitous in human life. Social events – either natural disasters or man-made accidents – would potentially prime mortality concerns (Coleman et al., 2017). To cope with mortality salience, consumers may exhibit a myriad of behavioral responses (Schindler et al., 2013; Sarial-Abi et al., 2017). Prior research has shown that increasing consumption activities serves as a means of neutralizing the death-related trepidation (e.g., Mandel and Smeesters, 2008). For example, consumers exposed to mortality salience tend to spend large amounts of money on a wide range of items such as food and clothing (Kasser and Sheldon, 2000; Ferraro et al., 2005), and are more likely to immediately consume, with less deferral (Coleman et al., 2017). However, other literature has documented that mortality salience can result in keeping wealth preserved or transferring possessions to future generations, a seemingly opposite behavioral pattern of indulgent consumption (Erikson, 1980; Price et al., 2000). These inconsistent findings suggest that mortality salience does not unconditionally augment overconsumption tendencies. We add to this research by introducing a potential moderator that may help to resolve this puzzle: a belief in karma.

Karma, the meta-ethical doctrine of causation, suggests that individual actions – both good and bad – give rise to positive and negative outcomes, sometimes in this life or in the hereafter (Converse et al., 2012; White et al., 2017). Consumption becomes excessive when it exhausts consumers’ mental or financial resources, thus negatively affecting personal and collective well-being (Sheth et al., 2011; Herziger et al., 2017). As noted by Pace (2013) and Mick (2017), excessive consumption is closely associated with destroying natural resources, encouraging decadent lifestyles, and breeding social inequality. Since these undesirable consequences essentially go against the belief system (Kulow and Kramer, 2016), we propose that belief in karma is an important qualification on the facilitating effect of mortality salience on excessive consumption (Ferraro et al., 2005; Mandel and Smeesters, 2008). Overall, we show that the effect of mortality salience is moderated by the individual’s belief in karma. For those who have a weak karmic belief, making mortality salient leads to a greater preference for excessive consumption. Conversely, for those who hold a strong belief in karma, the reverse emerges.

This paper is organized as follows. To begin with, we offer an overview on mortality salience, karmic belief, and overconsumption propensity to provide a theoretical basis for hypotheses. We then demonstrate how mortality salience and belief in karma jointly determine consumer excessiveness (study 1). This is followed by a section that examines the mediating role of temporal perspective, aiming to uncover the process by which the effect might occur (study 2). Next, we identified a boundary condition (i.e., benefit appeal) of the interaction effect (study 3). Finally, we conclude with theoretical and practical contributions of this research.

## Theory and Hypotheses

### Terror Management Theory and Karmic Belief System

Terror management theory (TMT) suggests that events reminding individuals of death engender overwhelming existential insecurity (Schindler et al., 2013). To buffer this death terror, people feel inclined to adopt an approach – psychically or physically – to help cope with the death-related threats. Substantial research has shown that consumers faced with an impending death tend to increase consumption quantities (Kasser and Sheldon, 2000; Ferraro et al., 2005; Mandel and Smeesters, 2008). In other word, thoughts of death might prompt people to indulge in various consumption regardless of the consequences.

Often referred to as “ethical principle of causation,” karma has played a vital role (e.g., serving as personal guidelines) in the social life (Pace, 2013). A nascent stream of research has indicated that belief in karma does affect consumers’ decision-making (Kopalle et al., 2010; Kulow and Kramer, 2016). In general, two important tenets characterize doctrines of karma: reincarnation and the doctrine of causation that good/bad deeds lead to good/bad outcomes (Kopalle et al., 2010; White et al., 2017). Reincarnation, the first tenet, functions as the bridge between individual’s this life and the hereafter (White et al., 2017). The second is about the nature of individual’s actions, where we can roughly classify them into good (appropriate) and bad (inappropriate). That is, karmic belief system declares that good deeds generate positive outcomes while bad deeds cause negative outcomes in the coming days (Kulow and Kramer, 2016). In the present research, however, we were not concerned with the functions of reincarnation. We instead concentrate on the causation doctrine because most people – both in the Eastern and Western society – are familiar with the karmic tenet that “the universe” rewards virtues and punishes transgressions (Converse et al., 2012).

### Mortality Salience, Karmic Beliefs, and Excessive Consumption

As discussed earlier, mortality salience has been associated with increased consumption (Ferraro et al., 2005; Mandel and Smeesters, 2008). Prior research has argued that individuals strive to live up to the standards upon which their self-esteem is based (Pyszczynski et al., 2004; Ferraro et al., 2005). When mortality is made salient, consumers will be more likely to engage in indulgent consumption (e.g., overeating or overspending) to alleviate mortality anxiety. Other literature has also suggested that increasing consumption serves as a trepidation buffer against potential demise. Mortality salience can generate negatively biased evaluations for future objects or events (Hermann et al., 2004), thus prompts consumers to actively make a quick choice, rather than delay through deferral (Coleman et al., 2017). To date, however, relatively little attention has been paid to the resultant evaluation of excessiveness. In this research, we reckon that an individual’s evaluated outcomes of excessive consumption should be taken into account because overconsumption has detrimental effects on personal and economic well-being (Herziger et al., 2017).

Given the doctrine of causation (i.e., good/bad deeds lead to good/bad outcomes) in karmic system, we argue that belief in karma is a key indicator for excessive consumption propensity (Kulow and Kramer, 2016). People can, arguably, restrain themselves from excess that may discourage fate’s favor in the future. In contrast, people with a weak belief in karma tend to have a short-term view of life (Kopalle et al., 2010). They are less likely to focus on the negative consequence of overconsumption (e.g., generating extreme environmental problem) in the long term. Put another way, retribution of virtue and vice can be an impersonal force that tracks moral behavior. Concerning excessive consumption runs counter to this principle of karmic values, we assume that belief in karma will moderate the mortality salience effect on overconsumption propensity (Converse et al., 2012; Mick, 2017). Taken together, belief in karma should lead individuals to strengthen the “good deed–good outcome” associations, and thus avoid “doing wrong” and counteract the tendency of unrestrained consumption triggered by mortality salience. Thus, we hypothesize:

Hypothesis 1. Belief in karma will moderate the effect of mortality salience on consumer preference for excessiveness, such that consumers experiencing mortality salience will be more likely to engage in excessive consumption when they hold a weak (vs. strong) belief in karma.

### Mediating Role of Temporal Perspective

In addition to examining the aforementioned effect, we seek to understand why mortality salience increases vs. decreases excessive consumption depending on belief in karma. We suggest that temporal perspective as a mediator underlying the joint effect. Temporal perspective, namely, the time horizon of an individual, bears effects on daily choices (e.g., basic diets) and personal lifestyles (Warin et al., 2015; Jarosz, 2018). Carney and Patrick (2017) suggest that such time perspective frames do play a role in health goals that people pursue (e.g., reduce tobacco usage). Importantly, a person with a long-term horizon (i.e., future-oriented) will pay more attention to the consequences in the distant future, even in the next life (Kopalle et al., 2010; Mello et al., 2016). The lessened emphasis on long-run outcomes, in turn, should increase the irrational and intemperate behavior for the purpose of being satisfied at present. Studies of gratification deferral also support this idea, with long-term orientation consumers delaying instant rewards and looking forward to the future benefits (Griskevicius et al., 2011; Jarosz, 2018).

Having a long-term perspective of time, of course, is fundamental to karmic beliefs, in that transmigrations and cause-and-effect retributions, as noted earlier, highlight corresponding outcomes in the long-range time (Converse et al., 2012; Pace, 2013). Most excessive consumption (e.g., indulgent consumption) is reported as shortsighted with respect to the consequences that materialize in the future (Mick, 2017; Hemetsberger, 2018). To illustrate, Kopalle et al. (2010) expound on the impact of karmic beliefs on consumer expectations, stating that a stronger belief in karma render consumers less prone to “strategically” lower their expectancy for temporarily feeling happier and more satisfied at present, regardless of the consequences in the future. For those believing in karma, they emphasize intertemporal connections that tie their past, present, and future as an unbroken continuum (White et al., 2017). In the proposed research, we utilize the perspective structure, to further comprehend the process by which the extent of karmic beliefs leads to distinct patterns of excessive consumption. We conjecture the interaction effect in hypothesis 1 will be driven by individuals’ temporal perspective. We therefore hypothesize:

Hypothesis 2. Temporal perspective will mediate the interactive effect of mortality salience and belief in karma on excessive consumption.

### Qualification and Constraints

Although these hypotheses seem straightforward, there are constraints on their applicability. Considerations for self and others are widely recognized and discussed in terms of evaluation of behavioral consequences (McCann et al., 2010; Kulow and Kramer, 2016; Schlosser and Levy, 2016). From the perspective of oneself, for one thing, overconsumption is well known to bound up with defective health status (e.g., morbid obesity). For another, consumer excessive activities can also result in environmental problem, resource scarcity, and social inequity in the community (Hakansson, 2014; Mick, 2017). Hence, individuals may reduce excessive consumption for egoistic and altruistic reasons, and advertising appeals may highlight self-benefit and other-benefit (Fisher et al., 2008; White and Peloza, 2009). The existing work has extensively investigated conditions under which appeals framed as self-benefit vs. other-benefit result in relatively more consumer responses; however, there is no consensus as to which appeal type is generally more persuasive. Although people from different cultural contexts may respond differently to two types of appeals (e.g., Zhang and Gelb, 1996), an emphasis on self or other in the appeals can provisionally invoke consumers’ egoistic or altruistic motivation (Peloza et al., 2013), subsequently affecting their behavior. In this research, we propose that self-benefit (vs. other-benefit) appeal may attenuate the association between mortality salience and belief in karma on excessive consumption propensity.

Karmic beliefs consider not only the valance of ones’ actions but also individuals’ moral and psychological reasons for engaging in those actions. Prior research suggests that karmic beliefs are more strongly associated with altruistically motivated behavior (Kulow and Kramer, 2016). Egoistically motivated acts (e.g., avoiding excessiveness), to some extent, represent selfish acts that may engender karmic punishments (Converse et al., 2012). That is, if the underlying motivation for a moderate act (e.g., reducing overconsumption) is perceived as self-benefiting because an appeal cues self-benefit, then the resulting karmic consequences is less likely to be positive. Focusing on self-benefit promotes benefit to oneself over benefit to others or the environment (Steg et al., 2014; Schlosser and Levy, 2016). Given the possibility of realizing a self-benefiting from avoiding overconsumption, individuals with a strong (vs. weak) belief in karma may thus response less favorably to appeals that cue self-benefit because the requested acts will no longer qualify as unselfish one, and hence not engender karmic rewards. Thus, the proposed interaction effect of mortality salience and belief in karma on excessiveness will be attenuated when cuing self-benefit. More formally:

Hypothesis 3. The interaction effect between mortality salience and belief in karma on excessive consumption will be eliminated for consumers under the self-benefit (vs. other-benefit) appeal.

### Overview of Current Studies

Three studies were conducted to test the hypotheses. Study 1 examined the differential impact of mortality salience on excessive consumption among participants varying in karmic belief strength. That is, mortality salience will interact with belief in karma to affect excessive consumption, supporting hypothesis 1. Study 2 provided an evidence that consumers’ temporal perspective will mediate the main effect, validating hypothesis 2. Study 3 replicated and extended the findings of previous studies. We identified a boundary condition for the interaction effect, showing that the effect will only occur when individuals focus on other-benefit, verifying hypothesis 3.

## Study 1

The core objective of study 1 is to investigate how consumers’ belief in karma affects their preference for excessive consumption when they experience mortality salience. The study included two manipulated factors (i.e., mortality salience and belief in karma). We anticipated that consumers with weaker belief in karma to respond more favorably to excessive consumption when confronted death reminder, as compared to those in the face of dental pain.

### Methods

#### Participants and Design

A total of 132 graduate students (*M*_age_ = 22.39; 51.5% males) at a large public university in China participated in this experiment in exchange for monetary compensation. The statistical power was computed using G^∗^Power 3.1 software (Faul et al., 2009). A sensitivity analysis with a significance level of 0.05, a statistical power 1-*β*, and a sample size of 132 revealed that effect size (*f*) is 0.25. On entering the lab, participants were randomly allocated to different cells, sitting at individual cubicles for a private space. More specifically, participants were randomly assigned to four conditions of a 2 (mortality salience: death vs. dental pain) × 2 (karma prime: absent vs. present) between-subjects design.

#### Procedure

First, mortality salience was manipulated by a writing task. According to Sarial-Abi et al. (2017), we instructed participants to write a 200-character paragraph about either death or dental pain. In the death condition, participants wrote an essay in response to this prompt: “Please think about your own death. Then, write a paragraph to describe how you feel AND what you would do once you are physically dead.” The participants in the other condition, instead, completed a narrative task following this instruction: “Please recount your experience about toothache. Then, write a paragraph to express how you feel AND what you would do when facing your dental pain.” Prior to being asked to evaluate the level of mortality salience, participants were told to finish several filler tasks. Participants then responded to three statements on a 7-point scale ranging from 1 (*not at all*) to 7 (*very much*). The measure includes three items adopted from Ferraro et al. (2005) such as, “My worry about death is overwhelming (*α* = 0.86).”

Next, participants moved to a karma-priming task that consisted of two parts: assessing a video commercial and doing a reading task (Kulow and Kramer, 2016). Specifically, participants in the karma-present condition were asked to view a short commercial that centered on karmic beliefs: what goes around comes around. The scenario began with a man littering, touching off a series of events through several others and, eventually, circling back to him (i.e., the original man) with negative outcomes (being struck by an arrow and getting knocked down by a truck). The tagline “It all comes back to you” was presented in the end of the commercial. In the karma-absent priming condition, participants viewed a karma-irrelevant commercial about a garbage classification. It featured some individuals picking unsuited sites to do sports, like playing golf in a basketball court and playing basketball in a football field. The commercial video ended with the slogan, “Pick the proper place for each category of rubbish.” Immediately after watching the video, participants evaluated on a seven-point scale (1 = *not at all*, 7 = *very much*) in terms of how humorous and how appealing it was. A pilot study among 67 individuals drawn from the same pool rated two commercials as equally humorous (*p* > 0.10) and appealing (*p* > 0.05).

Going forward, participants moved to the second part of karma-priming task (Kopalle et al., 2010). Specifically, those in karma-salient condition read a paragraph titled “Karma: You reap what you sow” that underlined the crucial tenets of karmic system, such as current actions lead to corresponding results in the future (e.g., being untruthful renders one utterly isolated), whereas those in the neutral condition read a passage titled “Life: Full of routine activities,” narrating regular events (e.g., calling friends) on an ordinary day. Participants were then told to write down an example consistent with the core idea of either karma-focused or routine-focused passage. See Supplementary Appendix A for more details. Participants next reported their karmic belief strength by rating the four statements such as, “Doing evil causes negative outcomes in this life or in the hereafter,” and “Good actions at present cause good outcomes in futures” (Kopalle et al., 2010; 1 = *strongly against*, 7 = *strongly favor*; *α* = 0.84).

After that, participants reported their likelihood of excessive consumption. We created the index by computing the average score of four items adapted from Hakansson (2014) and Herziger et al. (2017): “I will spend my money regardless of consequences;” “I will make a purchase as much as possible”; “I will buy what I want casually”; “I will do shopping according to my real need [reverse-coded]” (*α* = 0.84; 1 = *not likely at all*, 7 = *very likely*). Finally, to evaluate the alternative accounts on the proposed effect, participants responded to additional items capturing potential constructs (i.e., mood state, materialism, self-esteem). Specifically, participants responded their agreement on respectively 10 positive items (e.g., “excited”; *α* = 0.84) and 10 negative items (e.g., “hostile”; *α* = 0.73) that captured mood state (Watson et al., 1988). Three indices measuring materialism (e.g., “To what extent I gain happiness through my possessions,” *α* = 0.74; Kasser and Sheldon, 2000) and three items measuring self-esteem (e.g., “I hope I can earn more respect,” *α* = 0.71; Richins, 2004) were anchored on scales of 1 (*not at all*) to 5 (*very much so*). See Supplementary Appendix B for all measures. We also collected participants’ demographic information. Upon finishing the experiment, participants were thanked, debriefed, and paid.

### Results

#### Manipulation Check

We developed an average score of the three questions that served as a check for mortality salience. Validating the manipulation, a 2 × 2 ANOVA result yielded only the expected main effect of mortality salience: Participants in the death condition felt mortality more salient than those in the dental pain condition [*M* = 4.96 vs. 3.94, respectively; *F*(1,128) = 13.31, *p* < 0.001; η^2^ = 0.09]. A similar analysis revealed only a main effect of karma prime, such that those in the karma-present condition had a stronger belief in karma than did those in the karma-absent condition [*M* = 4.79 vs. 4.01, respectively; *F*(1,128) = 8.87, *p* < 0.01; η^2^ = 0.07], confirming the success of the karma manipulation.

#### Excessive Consumption

**FIGURE 1:**
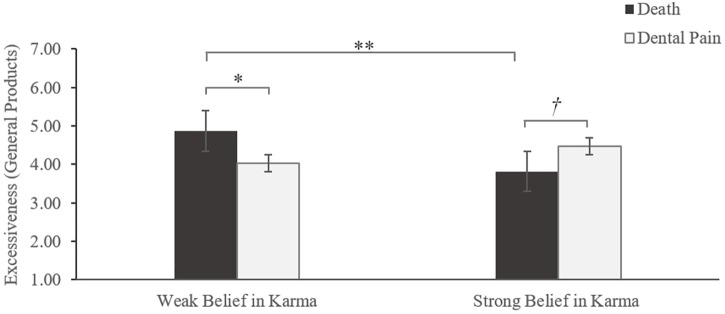
Study 1 results: the effect of mortality salience and belief in karma on consumer excessiveness (general products). *^†^p <* 0.1, ^∗^*p <* 0.05, ^∗∗^*p <* 0.01.

An ANOVA with mortality salience, karma prime, and their interaction as the independent variables, and overconsumption likelihood as the dependent variable yielded the expected significant interaction effect of mortality salience and belief in karma [*F*(1,128) = 9.46, *p* < 0.01; η^2^ = 0.07]. Neither main effect of mortality salience nor belief in karma was significant (*ps* > 0.20). Supporting our hypothesis 1, *post hoc* contrasts using test of simple effect further revealed that participants faced with death expressed more desirability for overconsumption than did those in the control condition when they were situated in the karma-absent prime [*M* = 4.87 vs. 4.03, respectively; *F*(1,128) = 6.03, *p* < 0.05], whereas participants responded less favorably to excessiveness in the face of mortality when they were primed present karma [*M* = 3.82 vs. 4.47, respectively; *F*(1,128) = 3.60, *p* < 0.10]. In addition, those in the karma-absent (vs. karma-present) prime showed greater preference for excessiveness when mortality is made salient [*M* = 4.87 vs. 4.03, respectively; *F*(1,128) = 8.90, *p* < 0.01]. No significant difference emerged in the dental pain condition between strong and weak karma believers for excessive consumption likelihood [*M* = 4.03 vs. 4.47, respectively; *F*(1,128) = 1.74, *p* > 0.10]. Figure 1 illustrates these results.

#### Alternative Accounts

To account for possible alternative explanations, we conducted a series of ancillary analyses. Separate ANOVAs revealed that neither main effects of mortality salience (*p* > 0.40) and belief in karma (*p* > 0.20) nor their interaction (*p* > 0.80) impacted positive affect. Negative affect was marginally higher in the death condition vs. the dental pain condition [*F*(1,128) = 3.24, *p* < 0.10] but was not affected by belief in karma (*p* > 0.40) or the interaction (*p* > 0.50). Including these variables as covariates did not dilute the focal two-way interaction (*p* < 0.01), which suggested that mood state did not account for the confirmed effect. We employed the same method to verify that our effect cannot be explained by materialism and self-esteem. We consistently found the same results about these constructs and we will not discuss them in any of the subsequent studies. See Supplementary Appendix C for all the statistical analyses aimed to rule out alternative explanations.

### Discussion

The results in study 1 validated our conceptualization. In support of hypothesis 1, compared with consumers exposed to dental pain, participants experiencing mortality salience will be more likely to engage in excessive consumption when they hold a weak belief in karma. However, when having a strong belief in karma, participants faced with death threat will tend to decrease overconsumption. Note that karma does not affect excessive consumption without mortality salience. This is also in keeping with the self-esteem effect, which only shows up after a morality prime (e.g., Ferraro et al., 2005). Furthermore, the alternative explanations of mood, materialism, and self-esteem were not supported.

## Study 2

The objectives of study 2 were twofold. First, we provided evidence to validate our proposed process, such that the effect of mortality salience and belief in karma on excessive consumption should be driven by temporal perspective. Second, we strived to test the robustness of the effects of study 1 by narrowing our attention to a specific product – cigarettes (Cherukupalli, 2010). Additionally, study 2 measures, rather than manipulates, participants’ karmic beliefs, focusing on the trait level – a person’s typical or average degree of belief in karma. Therefore, the study included one manipulated factor (mortality salience: death vs. dental pain) and one measured factor (belief in karma, continuous).

### Methods

#### Participants and Design

To collect the qualified samples (i.e., smokers), the respondents were first screened through two questions: “Do you smoke?” and “Have you quit smoking?” Non-smokers or smoke quitters were thus removed. One hundred and seventy-seven participants (*M*_age_ = 35.71; 77.40% males) were successfully recruited in the study for monetary compensation. The statistical power was computed using G^∗^Power 3.1 software (Faul et al., 2009). A sensitivity analysis with a significance level of 0.05, a statistical power 1-*β*, and a sample size of 177 revealed that effect size (*f*^2^) is 0.05. Eight responses were excluded because participants incorrectly answered the attention-filter question.

#### Procedure

Under the cover story that they would complete a series of unrelated studies, participants were first asked to complete the task of mortality salience manipulation and the instructions closely paralleled that used in study 1. Specifically, participants were randomly assigned to either death condition or dental pain condition to write a short passage. They then were required to complete the Belief in Karma Scale (*α* = 0.80) adopted from Kopalle et al. (2010). Next, four statements capturing participants’ temporal perspective (e.g., “I look forward to my future,” “I only plan for the short term [reverse-coded]”; Mello et al., 2016; *α* = 0.76) were measured using seven-point Likert items (1 = *strongly against*, 7 = *strongly favor*).

Afterward, participants reported the likelihood of excessive consumption for cigarette (e.g., “I will smoke regardless of consequences”; 1 = *very unlikely*, 7 = *very likely*; Herziger et al., 2017; *α* = 0.72), which was our dependent variable. Participants also indicated the degree to which they agreed to items (e.g., “I keep thinking about how short life really is”; 1 = *strongly against*, 7 = *strongly favor*; Ferraro et al., 2005; *α* = 0.79) that served as a manipulation check for mortality salience. Then, participants were probed for their suspicion on the purpose of our investigation, but none could guess the aim correctly. At the end, all participants answered standard demographic questions and the attention-filter question. Upon completion, participants were thanked and paid.

### Results

#### Manipulation Check

As expected, participants felt mortality more salient in the death vs. dental pain condition [*M* = 4.96 vs. 4.39, respectively; *F*(1,175) = 10.99, *p* < 0.001; η^2^ = 0.06], thereby confirming the manipulation of mortality salience.

#### Excessive Consumption

**FIGURE 2:**
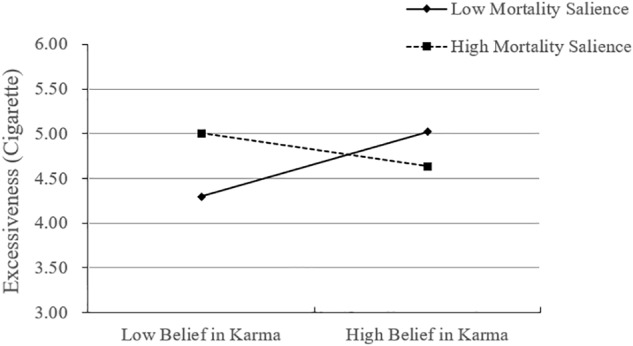
Study 2 results: the effect of mortality salience and belief in karma on consumer excessiveness (cigarettes).

A regression analysis with excessive consumption likelihood of cigarette as dependent variable and mortality salience, belief in karma, and their interaction term as predictors yielded a significant interaction between mortality salience and karmic belief strength (β = -0.19, *SE* = 0.05, *t* = -3.51, *p* < 0.001). Neither the main effect of mortality salience nor belief in karma (*ps* > 0.18) was found to significantly predict excessive consumption. To explore the significant interaction, we investigated excessive consumption between the mortality conditions at each level of karmic belief (mean ± 1 *SD*). For those who had a weak belief in karma (i.e., those who were 1.26 *SD* below the mean), there was positive effect of mortality salience such that making mortality salient leads participants to engage in excessive consumption (*β* = 0.23, *SE* = 0.09, *t* = 2.40, *p* < 0.05). Conversely, for those who had a strong belief in karma (i.e., those who were 1.26 *SD* above the mean), the reverse emerged (*β* = -0.25, *SE* = 0.10, *t* = -2.57, *p* < 0.05). These results supported our hypothesis 1, as shown in Figure 2.

#### Mediation Analysis

**FIGURE 3:**
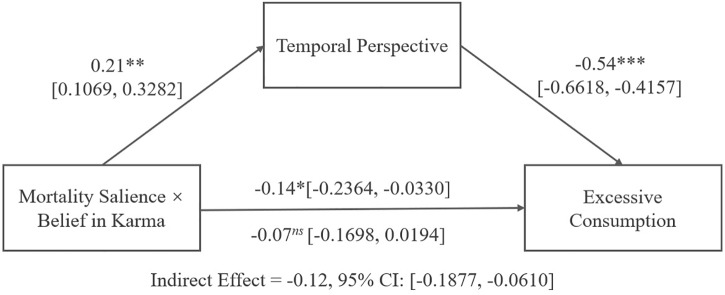
Study 2 results: mediation analysis with temporal perspective as a mediator. *^ns^p* > 0.05, ^∗^*p* < 0.05, ^∗∗^*p* < 0.01, ^∗∗∗^*p* < 0.001.

Following a mediated moderation approach, we next ran a mediational analysis using the PROCESS SPSS macro (Model 8; Zhao et al., 2010; Hayes, 2013). In the regression model, the dependent variable was excessive consumption (continuous), while the independent variables were mortality salience (death vs. dental pain), belief in karma (continuous), and temporal perspective (continuous). The effect of the mediator, temporal perspective, was significant (*β* = -0.54, *SE* = 0.06, *t* = -8.64, *p* < 0.001, 95% CI: [-0.6618, -0.4157]). The interactive effect of mortality salience and belief in karma on temporal perspective also reached significance (*β* = 0.21, *SE* = 0.05, *t* = -3.88, *p* < 0.01, 95% CI: [0.1069, 0.3282]). Most importantly, a bootstrap analysis confirmed a significant indirect effect at the highest order interaction (*β* = -0.12, *SE* = 0.03, 95% CI: [-0.1877, -0.0610]). Thus, mortality salience decreased temporal perspective for individuals with weak belief in karma, increasing excessive consumption. However, for those with high belief in karma, mortality salience increased temporal perspective, mediating the positive effect of mortality salience on excessive consumption. Collectively, these results support the notion that temporal perspective mediates the interactive effect of mortality salience and belief in karma on excessive consumption, thereby validating the hypothesis 2. Figure 3 displays the complete path coefficients.

### Discussion

Replicating the results of study 1 and extending them to preference for a specific consumption object (i.e., cigarette), we again observed evidence for the interactive effect of mortality salience and belief in karma on excessive consumption. As in study 1, belief in karma does not generally decrease excessive consumption, but only when mortality is made salient. Moreover, we confirmed the proposed mechanism driving the effect. That is, for those with weak belief in karma, mortality salience led to lower temporal perspective, which in turn contributed to higher likelihood of excessiveness. For those with a strong belief in karma, mortality salience, conversely, led to higher temporal perspective, thereby reducing the excessiveness intention.

## Study 3

In study 3, we attempted to replicate and extend our basic findings observed in studies 1 and 2. For this purpose, study 3 introduced a series of changes in procedure. First, we used a different dependent variable, red meat consumption. Second, we sought evidence, once again, for temporal perspective process explanation by altering the measure sequence for mediator. Third, study 3 explored whether the interactive effect of mortality salience and belief in karma on overconsumption propensity differ across benefit appeals. That is, we expect the confirmed effect to occur primarily among participants under other-benefit rather than self-benefit condition.

### Methods

Participating for one course credit, 230 undergraduate students at a public university in China were recruited. Four participants did not fully complete the experiment and were thus omitted from the data set, yielding a final sample of 226 (*M*_age_ = 21.42; 43.4% males). The statistical power was computed using G^∗^Power 3.1 software (Faul et al., 2009). A sensitivity analysis with a significance level of 0.05, a statistical power 1-*β*, and a sample size of 132 revealed that effect size (*f*) is 0.19. Upon arrival at the lab, participants were randomly assigned to conditions in a 2 (mortality salience: death vs. dental pain) × 2 (belief in karma: absent vs. present) × 2 (benefit appeal: self-benefit vs. other-benefit) between-subjects design.

First, we manipulated mortality salience and belief in karma. The instructions were closely paralleled those used in study 1, but we exchanged the two manipulations’ order. After responding to statements from two sets of seven-point scales (1 = *strongly against*, 7 = *strongly favor*) serving as manipulation check for the factors, mortality salience (*α* = 0.84) and belief in karma (*α* = 0.83), participants were told to complete several attention-filter questions.

Next, we utilized the modified excerpts of articles about excessive consumption of red meat in Hong Kong from the news publication *China Daily*, highlighting either self-benefit or other-benefit by emphasizing message relevant to core fonts of benefit. Specifically, in the self-framing condition, participants read the article titled “Being top carnivores no reason for Hong Kong to celebrate,” in which the author pointed out “excessive consumption of meat, especially the red variety, is known to cause health problems” and illustrated this point with “clinical obesity, heart disease, and colorectal cancer...” The headline and overall layout of news report (i.e., font, illustration, word count) were identical in the two conditions, with the exception of underlined narration in the other-considered condition where the author claimed that excessive red meat consumption “raises serious environmental issues and does harm to others” and gave several examples of this opinion, such as water shortages, greenhouse gases, and unsustainable land use (see Supplementary Appendix A for more details on the manipulation). Going forward, participants rated the extent to which the appeals were perceived as altruistic [reverse-scored] or egoistic (Kulow and Kramer, 2016; *r* = 0.59, *p* < 0.001). In both appeal conditions, questions were designed to measure participants’ trustworthiness of the report (*α* = 0.69) and expertise about red meat (*r* = 0.56, *p* < 0.001). We collected these data using a seven-point scale (1 = *not at all*, 7 = *very much*). They might affect the interaction effect and thus were collected.

Rather than administering the measure of temporal perspective after participants reported excessiveness intention as in study 2, study 3 measured temporal perspective before participants worked on consumption patterns. By doing so, we further provided evidence for our proposed process. Specifically, four items (e.g., “I look forward to my long-term future”; Mello et al., 2016; *α* = 0.80) were used to capture participants’ temporal perspective. After that, we used the statements similar to the items from study 1 but specified the products as red meat (e.g., “I consume red meat regardless of consequences,” “I consume red meat even if I don’t need it[reverse-coded]”; Herziger et al., 2017; *α* = 0.74) to collect participants’ excessiveness propensity. See Supplementary Appendix B for measures. Finally, participants indicated their gender and age, following which they were debriefed and thanked.

### Results

#### Manipulation Check

Participants’ response to the three manipulation check questions for mortality salience were averaged to form a manipulation check score. An ANOVA indicated a main effect of mortality salience [*F*(1,218) = 14.42, *p* < 0.001; η^2^ = 0.07], such that participants in the death condition felt more worried about mortality (*M* = 4.77) than those in the dental pain condition (*M* = 3.92). No other main effects or interactive effects reached significance (*ps* > 0.20). As for karmic belief manipulation, only a main effect of karma prime could be found [*F*(1,218) = 15.54, *p* < 0.001; η^2^ = 0.06], such that those in the karma-present condition had a higher belief in karma (*M* = 4.68) than did those in the karma-absent condition (*M* = 3.89). There were no other significant main effects or interactive effects (*ps* > 0.10). Thus, the manipulation of belief in karma was successful. A similar analysis revealed only a main effect of appeal type [*F*(1,218) = 41.91, *p* < 0.001; η^2^ = 0.12]. Results revealed that those in the other-benefit condition focused more on others (*M* = 4.93) than did those in the self-benefit condition (*M* = 3.84), confirming the success of the appeal type manipulation.

#### Excessive Consumption

We performed a 2 (mortality salience: death vs. dental pain) × 2 (belief in karma: absence vs. presence) × 2 (benefit appeal: self-benefit vs. other-benefit) between-subjects ANOVA on excessive consumption. The results yielded a three-way interaction among mortality salience, belief in karma, and appeal frame [*F*(1,218) = 6.99, *p* < 0.01; η^2^ = 0.03] along with a main effect of appeal frame [*F*(1,218) = 17.11, *p* < 0.001; η^2^ = 0.06], and a significant interaction between mortality salience and belief in karma [*F*(1,218) = 12.13, *p* < 0.001; η^2^ = 0.05].

**FIGURE 4:**
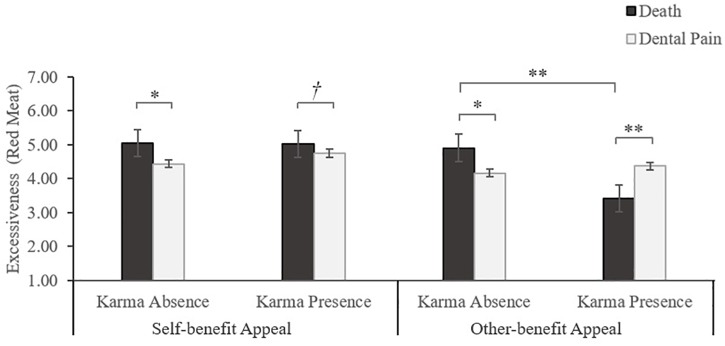
Study 3 results: the interaction effect on consumer excessiveness (red meat) in the self-benefit and other-benefit condition. *^†^p <* 0.1, ^∗^*p <* 0.05, ^∗∗^*p <* 0.01.

Next, we conducted a MANOVA to decompose the interaction in each benefit appeal. Under the self-benefit frame, preference for excessive red-meat consumption was significantly higher when faced with death vs. dental pain condition whether participants have karma absence [*M* = 5.04 vs. 4.44, respectively; *F*(1,221) = 4.62, *p* < 0.05] or karma presence [*M* = 5.02 vs. 4.75, respectively; *F*(1,221) = 1.68, *p* < 0.10]. Results in the other-benefit condition were similar to those in study 1. That is, karma-absent participants in the death condition expressed greater intention of red-meat excessiveness than did those in the dental pain condition [*M* = 4.91 vs. 4.17, respectively; *F*(1,221) = 6.76, *p* < 0.05]. In contrast, for karma-present participants, those in the death condition were less likely to engage in red-meat excessive consumption, compared to those in the dental pain condition [*M* = 3.41 vs. 4.37, respectively; *F*(1,221) = 5.10, *p* < 0.05]. Collectively, these results supported the hypothesis 3, as presented in Figure 4.

#### Mediation Analysis

To confirm the mediational role of temporal perspective, we then performed a mediation analysis using SPSS PROCESS macro (Model 12; Zhao et al., 2010; Hayes, 2013). In the regression model, excessiveness propensity (continuous) served as the dependent variable, and our independent variables were mortality salience (death vs. dental pain), karma prime (absence vs. presence), benefit appeal (self-benefit vs. other-benefit), and their interactions terms as predictors, including temporal perspective (continuous) as the mediator. Results indicated a significant effect of the mediator on excessiveness propensity (*β* = -0.41, *SE* = 0.06, *t* = -7.20, *p* < 0.001). The inclusion of participants’ temporal perspective in the model reduced the interaction significance of mortality salience × karma prime × appeal frame (*p* > 0.05). Most importantly, a bias-corrected bootstrap analysis revealed a significant indirect effect of the highest order interaction with temporal perspective as the mediator was statistically significant (*β* = -0.78, *SE* = 0.21, 95% CI: [-1.2414, -0.4267]). Thus, these results again established temporal perspective as a mediator, consistent with the findings from study 2.

#### Alternative Accounts

Lastly, we conducted the preceding analysis once again, including the potential constructs as covariates. Results revealed that participants’ trustworthiness (*p* > 0.10) and their expertise (*p* > 0.30) did not account for the effect.

### Discussion

In the other-benefit condition, we replicated the interactive effect on excessive consumption that we observed earlier. But under a self-benefit appeal, the asymmetry did not emerge, such that mortality salience increases consumer excessiveness whether they have a strong or weak belief in karma. Study 3 identified a boundary condition of the interaction effect of mortality salience and belief in karma, indicating that the interaction will be more robust when consumers highlighted other-benefit.

## General Discussion

Making mortality salient via social events (e.g., homicide) or natural events (e.g., tsunamis) is frequently accessible among individuals. One way in which they might cope with is through consumption (Mandel and Smeesters, 2008; Coleman et al., 2017). In the current research, we developed a novel, integrated framework with regard to how mortality salience, karmic beliefs, and benefit appeal interact to determine excessive consumption. Results from three studies confirmed our conceptual model and propositions. Foremost, mortality salience interacts with consumers’ belief in karma to affect their excessive consumption propensity. More importantly, we pinpoint a specific mechanism that underlies the effect. In addition, when consumers primed a self-benefit appeal, the confirmed effect no longer exists. Implications arise for both theory and practice.

### Theoretical Implications

Our research adds to the literature in several ways. First, we contribute to the extant literature on TMT. Although research on mortality salience (MS) effects is extensive (Arndt et al., 2004; Ferraro et al., 2005; Mandel and Smeesters, 2008; Rindfleisch et al., 2009; Hakansson, 2014; Coleman et al., 2017; Herziger et al., 2017; Mick, 2017), to our knowledge no research has considered consumers’ belief in karma and explored how it affected excessive consumption propensity after mortality salience. Considerable evidence has suggested that mortality salience promotes consumption behavior (e.g., Mandel and Smeesters, 2008), we provide evidence that this is not always the case and that the peculiar belief can play the crucial role. As we mentioned in Section “Introduction,” the inconsistent findings suggest that mortality salience does not unconditionally increasing consumption tendency (Price et al., 2000; Ferraro et al., 2005). We fill this gap by revealing a potential moderator and demonstrate that excessiveness propensity can be ameliorated by belief in karma after mortality is made salient.

Next, the research enriches literature on peculiar beliefs by showing that belief in karma manifests a causal link of current actions resulting in future consequences that ultimately impact consumers’ behaviors in the present. We take a new perspective on this factor (i.e., belief in karma) to identify one important boundary condition for mortality salience effects on excessive consumption (Kopalle et al., 2010; Kulow and Kramer, 2016). While Converse et al. (2012) previously pointed out that individuals with a strong belief in karma were hoping to positively influence a future outcome by engaging in prosocial acts (e.g., volunteering their time to social causes), our research suggests that excessive consumption triggered by mortality salience represents a bad deed that might generate bad outcomes and thus, was influenced by belief in karma.

Furthermore, at a deeper level, we extend the psychology literature by establishing the underlying mechanism (i.e., the temporal perspective) by which consumers engage in excessive activities in the face of mortality salience. While two established coping strategies, bolstering self-esteem and cultural worldview, are documented in the previous work (Mandel and Smeesters, 2008; Schindler et al., 2013), we seek to attach great importance to another explanation and explore the mediating role of temporal perspective.

At a very broad level, our research sheds light on how temporal perspective influence individuals’ decision-making (Kopalle et al., 2010; Warin et al., 2015; Carney and Patrick, 2017; Jarosz, 2018). We demonstrate that belief in karma may expand temporal perspective (i.e., future-oriented horizon) after mortality salience. Prior work has suggested that believing in karma helps bolstering long-term orientation, thus counteracts the tendency to lower expectations (Kopalle et al., 2010). In this research, we found that mortality salience leads to increasing likelihood of excessive consumption only for consumers with weak belief in karma due to the contracted time horizon. This is allied with the viewpoint that the temporal framing of a decision may affect consumer behavior (Malkoc and Zauberman, 2006).

Exploring the role of temporal perspective on excessive consumption also provides a unique opportunity to advance our understanding of construal level theory (CLT; Trope and Liberman, 2000). CLT has identified a host of consequences to changes in construal (Trope and Liberman, 2010). For example, events and objects construed at higher level lead to increased self-control (Fujita et al., 2006). Excessive consumption (e.g., shopping obsessiveness) could be regarded as difficulty in self-control (Mick, 2017). We extend these literatures and suggest that individual with a weak belief in karma are prone to excessive consumption when exposed to mortality salience.

Additionally, by exposing the boundary conditions (i.e., benefit appeal) for the interaction effect on excessive consumption, this research also enriches the repertoire of altruism literature. Study 3 results show that karmic beliefs no longer exert an influence when individual is self-focused by cuing self-benefits. Thus, our study contributes to research on altruism by empirically demonstrating the viewpoint that focusing on self-benefit (i.e., egoism) is negatively associated with ecological attitudes and sustainable behaviors (Steg et al., 2014; Schlosser and Levy, 2016).

### Practical Implications

This research also reveals practical implications for marketers. When mortality is made salient, it is crucial for marketers to identify the opportunity to reap the benefit. While prior work suggested that mortality salience causes more consumption (e.g., Arndt et al., 2004), we provide support for belief in karma as an individual difference variable that impacts the likelihood of engaging in excessive consumption after mortality salience. In particular, individuals who believe in karma, as compared to those who do not, should be more likely to do “right” things (Kulow and Kramer, 2016), such as avoiding overconsumption. Therefore, marketers could pay more attention to consumers’ belief in karma when mortality salience occurs. At the same time, our research offers insights into how marketers of hedonic products could employ consumers’ temporal perspective. As we demonstrated in studies 2 and 3, shortened temporal perspective would make it more likely that consumers engage in consuming products that are bought for pleasure rather than a functional purpose. Thus, companies could adopt some communication strategies (e.g., advertising programs) that reduce consumers’ future-oriented temporal perspective, which should, in turn, enhance consumers’ intentions to excessive consumption.

An additional practical implication of this research lies in the findings that benefit appeal eliminates the effects of mortality salience and belief in karma. We found that, in the self-benefit appeal, consumers tend to respond favorably to excessive consumption whether they believe in karma or not. Marketers can utilize these findings to create effective persuasion appeals targeted at specific products or services. This is also consistent with research in experimental economics on providing scientific information on a product’s utility (Zhao et al., 2013). When trying to elicit consumers’ purchase desire, companies should highlight egoism rather than altruism in persuasive appeals. Further, while the above techniques may help boost firm profits, public policy makers need to monitor whether the manipulation of benefit appeal is detrimental to consumer welfare (Sheth et al., 2011; Herziger et al., 2017). All in all, our results suggest that it would behoove marketers and public policymakers to understand the relationship among belief in karma, temporal perspective, and benefit appeal when mortality is made salient.

### Limitations and Future Research

Although our research makes a number of contributions, it also has several limitations that suggest a number of potentially future research opportunities. For example, study 2 collected a sample of 77.40% males and analyzed these data to validate our conceptualization. Though it is true that more males smoke in China, examining the impact of factors such as gender also seems potentially useful.

With regard to the key dependent variable, we only measured the propensity (i.e., likelihood) of excessive consumption. Future research could measure actual item selection or purchasing. It is also worth noting that, there are many forms of excessive consumption such as shopping obsessiveness and luxury fascination (Mick, 2017). Although our focus was on excessive consumption as operationalized by vice goods (e.g., studies 2 and 3), it might be applicable to other hedonic consumption settings (Cherukupalli, 2010; Jain, 2012).

Because people avoid negative-valenced consumption does not mean they will involve in positive-valenced counterpart, it would be intriguing to examine whether the current effects hold or disappear in the context of positively valenced consumption. For example, when exposed to mortality salience, would karma believers be more likely to engage in sustainable behavior such as recycling to be green (Kidwell et al., 2013)? What actions will consumers take if they are consuming reasonably good products, such as life insurance options or medical procedures (Coleman et al., 2017)? We call for more research on this topic.

Excessive consumption could be thought of as difficulty in self-regulation or impulse-control (Muraven and Baumeister, 2000; Mick, 2017). The relative availability of self-regulatory resources plays a critical role in indulgent vs. restrained eating behavior for females (Ferraro et al., 2005). Future research could explore how self-regulation or self-control affect the current effects.

Cultural context is an additional likely moderator of the confirmed effects. As study 3 indicates, the interaction between mortality salience and belief in karma on excessive consumption disappears when presenting self-benefit appeal. However, it is possible that self-benefit is a boundary condition more for Chinese participants. Prior research has shown that cross-cultural differences in the persuasive power of messages (e.g., Zhang and Gelb, 1996). For example, people in collectivistic cultures may find messages cuing other-benefit more persuasive. Thus, cultural factors are likely to present important boundary conditions for the confirmed effects, a possibility we would be eager to see future research address.

Finally, though we demonstrated the mediating role of temporal perspective in the current research (studies 2 and 3), there are other potential factors that could have influenced the confirmed effects as well. For example, could promotion or prevention orientation drive consumers to highlight reaping rewards or avoiding punishments (Ran et al., 2016)? Examining how exactly the above processes work also seems to be a fertile ground for subsequent research.

## Conclusion

This research has shown that mortality salience interacts with belief in karma to affect excessive consumption. When consumers have a weak belief in karma, those faced with mortality salience tend to engage in consumption excess than did those in the control condition, whereas consumers in the death condition are less likely to overconsume when they have a strong belief in karma. Moreover, consumers’ temporal perspective drives this effect. We also identified the boundary condition (i.e., benefit appeal) for the interaction between mortality salience and belief in karma. While contributing to the existing literature, these findings also suggest that managers could explore the factor (i.e., belief in karma) in their markets, and then adopt effective measures to respond to mortality salience triggered by various events such as homicide, and other such major disasters or crises. Understanding local preferences are important when it comes to the transcendental as well.

## Ethics Statement

This research was approved by the ethics committee of the School of Management, Jinan University, China. All participants gave their written informed consent before the experiments.

## Author Contributions

SC and HW conceived and designed experiments. SC and LM carried out the experiments and analyzed the experimental results. SC wrote the manuscript. YR edited the manuscript.

## Conflict of Interest Statement

The authors declare that the research was conducted in the absence of any commercial or financial relationships that could be construed as a potential conflict of interest.
