# Supplementary material for: Believing in Karma: The Effect of Mortality Salience on Excessive Consumption
Source: Front Psychol. 2019 Jul 8;10:1519. doi: 10.3389/fpsyg.2019.01519 (PMC6628939; doi:10.3389/fpsyg.2019.01519)
Supplement: Supplementary file 1 [file Table_1.DOCX]

**APPENDIX A**

The reading materials of karmic beliefs manipulation used in study 1.

A.1 The karma-present article


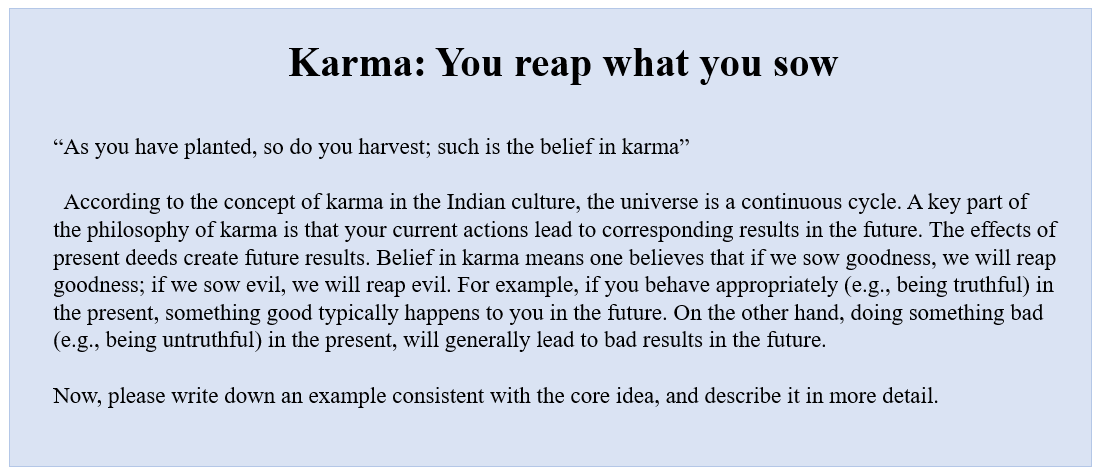


A.2 The karma-absent article


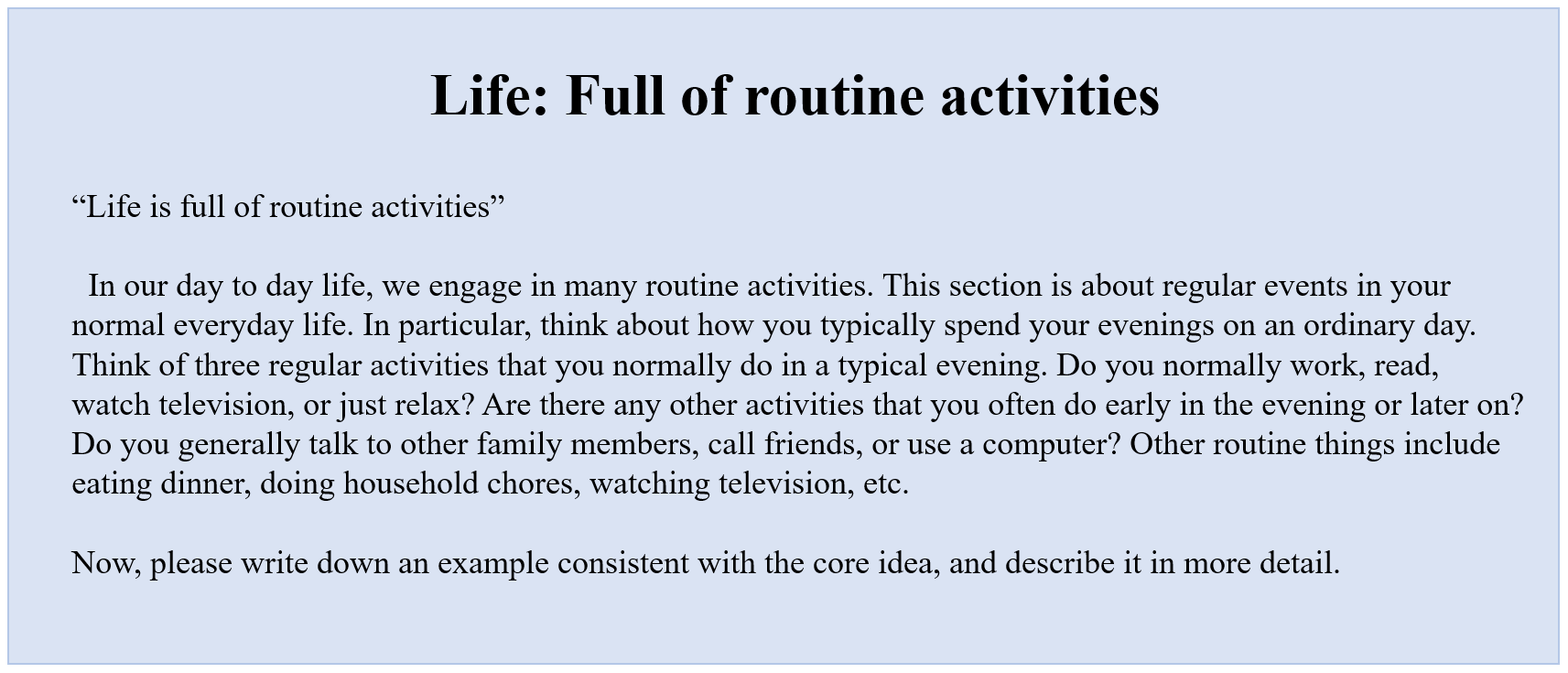


The materials of news report about meat overconsumption used in study 3.

B.1 Self-framing article


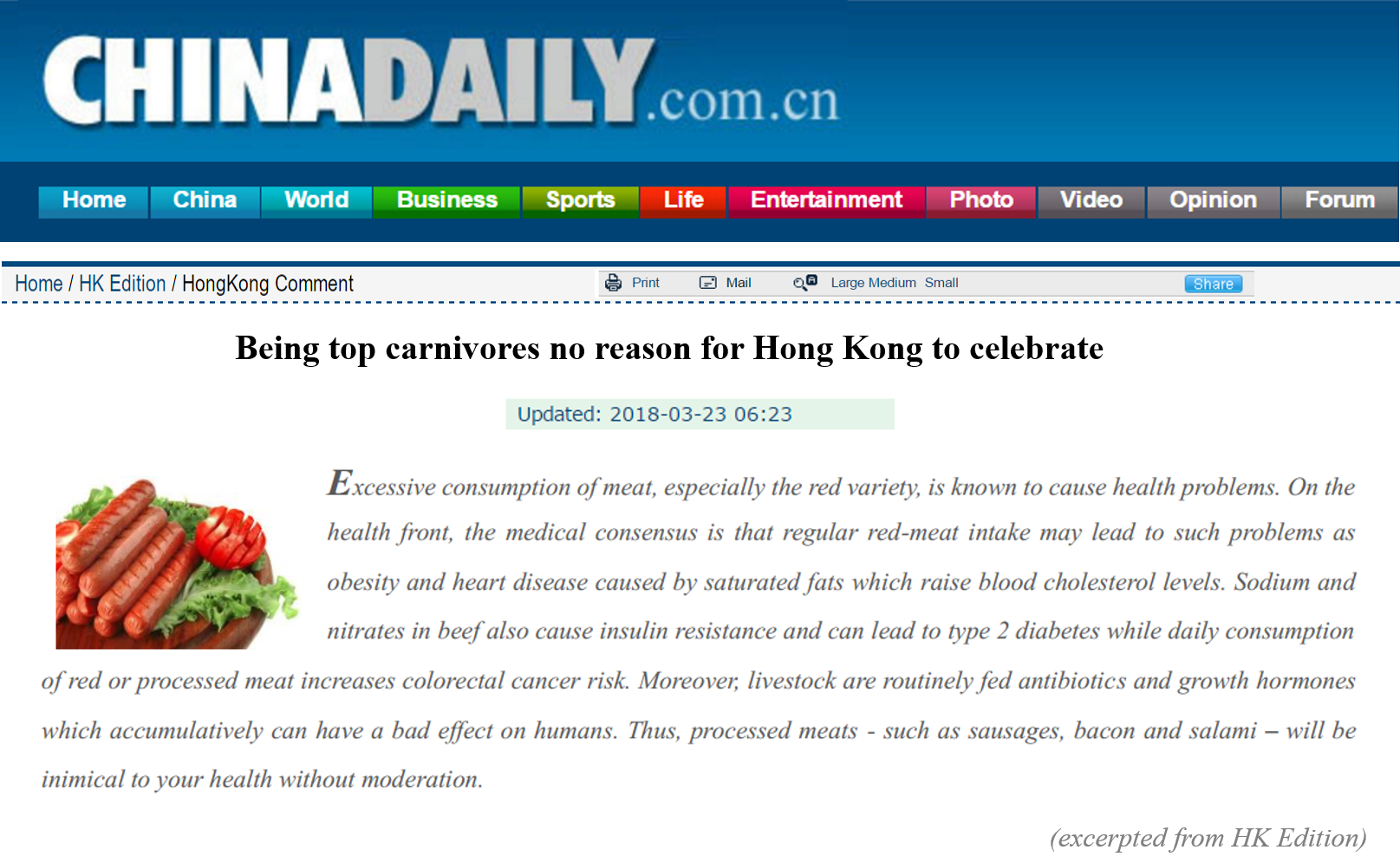


B.2 Other-framing article


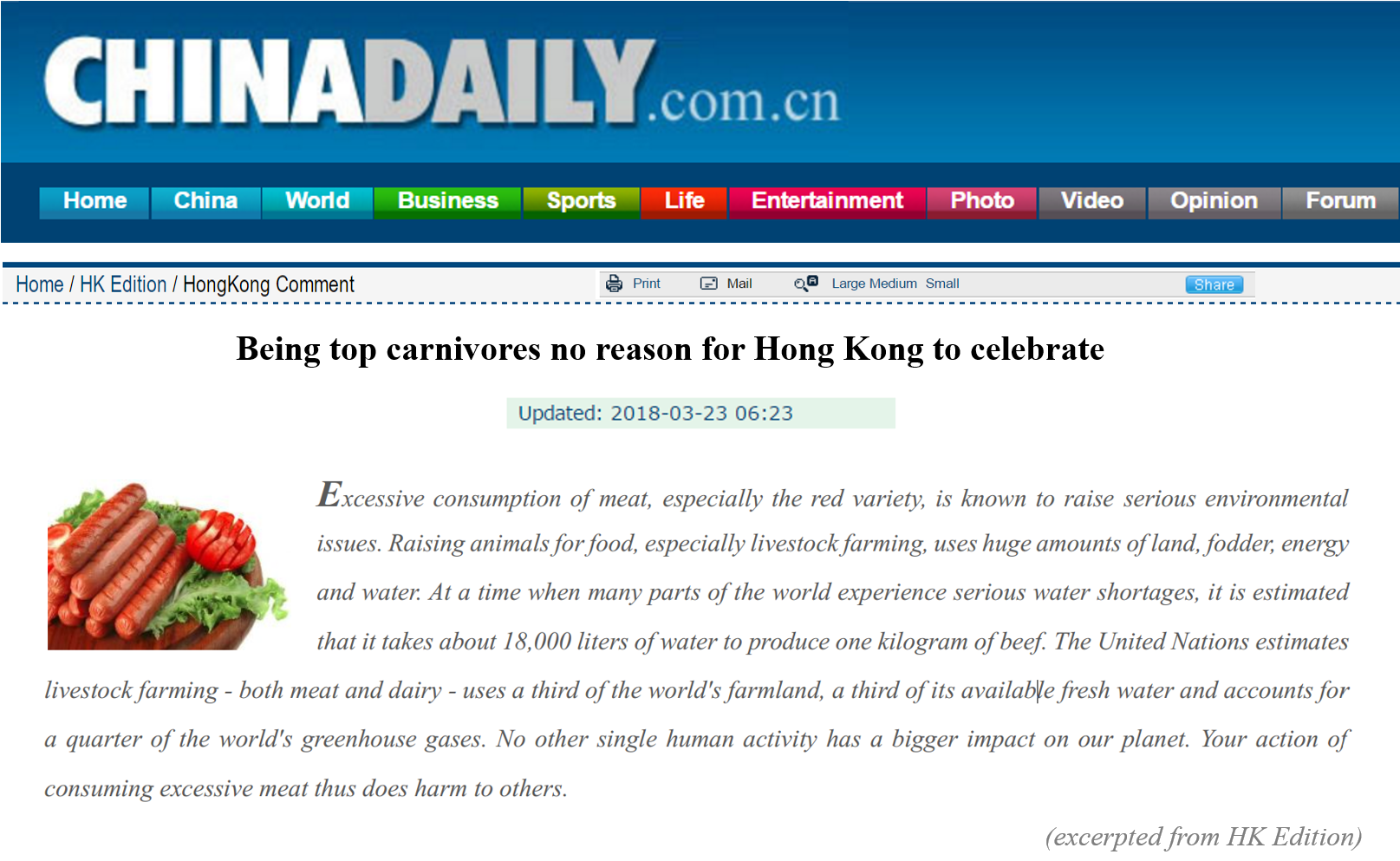


**APPENDIX B**

Table 1 | Measures in study 1.

| **KMO = 0.74, Bartlett sig*.* < 0.001, Explained Variance = 71.20%**  **Variables** | **Loading** |
| --- | --- |
| **Mortality Salience (*α* = 0.86; CR =0 .91; AVE = 0.76)** |  |
| I am afraid to die. | 0.88 |
| I often think about how short life really is.  My worry about death is overwhelming. | 0.87  0.87 |
| **Belief in Karma (*α* = 0.84; CR = 0.89; AVE = 0.68)** |  |
| Doing evil causes negative outcomes in this life or in the hereafter. | 0.83 |
| Good actions at present cause good outcomes in futures.  I believe in karma.  The universe is a continuous cycle. | 0.82  0.83  0.81 |
| **Excessive Consumption (*α* = 0.84; CR = 0.87; AVE = 0.63)** |  |
| I will make a purchase as much as possible. | 0.83 |
| I will buy what I want casually. | 0.80 |
| I will spend my money regardless of consequences.  I will do shopping according to my real need. | 0.84  0.71 |

Table 2 | Measures in study 2.

| **KMO = 0.78, Bartlett sig*.* < 0.001, Explained Variance = 61.51%**  **Variables** | **Loading** |
| --- | --- |
| **Mortality Salience (*α* = 0.79; CR =0 .88; AVE = 0.72)** |  |
| I am afraid to die. | 0.82 |
| I often think about how short life really is.  My worry about death is overwhelming. | 0.83  0.89 |
| **Belief in Karma (*α* = 0.80; CR = 0.86; AVE = 0.61)** |  |
| Doing evil causes negative outcomes in this life or in the hereafter. | 0.83 |
| Good actions at present cause good outcomes in futures.  I believe in karma.  The universe is a continuous cycle. | 0.72  0.77  0.80 |
| **Temporal Orientation (*α* = 0.76; CR =0.79; AVE = 0.50)** |  |
| I look forward to my future.  I have many plans for the future.  I don’t mind giving up today’s fun for success in the future.  I only plan for the short term. | 0.70  0.71  0.66  0.75 |
| **Excessive Consumption (*α* = 0.72; CR = 0.80; AVE = 0.51)** |  |
| I smoke as much as I want. | 0.75 |
| There is no need to control my smoking quantities | 0.69 |
| I smoke regardless of consequences.  I smoke according to my own desire no matter what it takes. | 0.67  0.72 |

Table 3 | Measures in study 3.

| **KMO = 0.75, Bartlett sig*.* < 0.001, Explained Variance = 67.75%**  **Variables** | **Loading** |
| --- | --- |
| **Mortality Salience (*α* = 0.84; CR =0 .90; AVE = 0.75)** |  |
| I am afraid to die. | 0.87 |
| I often think about how short life really is.  My worry about death is overwhelming. | 0.86  0.87 |
| **Belief in Karma (*α* = 0.83; CR = 0.89; AVE = 0.67)** |  |
| Doing evil causes negative outcomes in this life or in the hereafter. | 0.83 |
| Good actions at present cause good outcomes in futures.  I believe in karma.  The universe is a continuous cycle. | 0.81  0.83  0.79 |
| **Temporal Orientation (*α* = 0.80; CR =0.85; AVE = 0.59)** |  |
| I look forward to my future.  I have many plans for the future.  I don’t mind giving up today’s fun for success in the future.  I only plan for the short term. | 0.75  0.75  0.82  0.74 |
| **Excessive Consumption (*α* = 0.74; CR = 0.80; AVE = 0.51)** |  |
| I consume red meat as much as possible. | 0.59 |
| I consume red meat even if I don’t need it. | 0.76 |
| I consume red meat regardless of consequences.  I consume red meat casually.  **Interest Frame (*r* = 0.59, *p*< 0.001; CR = 0.87; AVE = 0.77)**  To what extent do you feel the appeal was perceived as altruistic  To what extent do you feel the appeal was perceived as egoistic | 0.80  0.68  0.89  0.87 |

**APPENDIX C**

Table 1 | The ANOVAs ruling out alternative explanations (studies 1 and 2).

| **Studies** | **Potential Constructs** | **Mortality Salience** | | **Belief in Karma** | **Interaction Item** |
| --- | --- | --- | --- | --- | --- |
| **Study1** | Positive affect | *F* =0.62, *p* =0.43 | | *F* =1.62, *p* =0.21 | *F* =0.03, *p* =0.87 |
|  | Negative affect | *F* =3.23, *p* =0.07 | | *F* =0.61, *p* =0.44 | *F* =0.46, *p* =0.50 |
|  | Materialism | *F* =4.35, *p* =0.04 | | *F* =0.47, *p* =0.49 | *F* =0.34, *p* =0.56 |
|  | Self-esteem | *F* =0.23, *p* =0.64 | | *F* =1.13, *p* =0.28 | *F* =1.41, *p* =0.23 |
| **Study2** | Positive affect | *F* =0.14, *p* =0.71 | | *F* =1.03, *p* =0.44 | *F* =0.72, *p* =0.81 |
|  | Negative affect | *F* =3.51, *p* =0.06 | | *F* =1.46, *p* =0.11 | *F* =0.76, *p* =0.76 |
|  | Materialism | *F* =1.52, *p* =0.22 | *F* =0.72, *p* =0.82 | | *F* =0.51, *p* =0.96 |
|  | Self-esteem | *F* =1.92, *p* =0.16 | *F* =0.74, *p* =0.79 | | *F* =0.75, *p* =0.77 |

Table 2 | Correlation matrix for the key variables (study 2).

| **Scale** | **1** | **2** | **3** | **4** |
| --- | --- | --- | --- | --- |
| 1.Mortality Salience | (0.79) |  |  |  |
| 2.Belief in Karma | 0.05 | (0.80) |  |  |
| 3.Temporal Perspective | 0.17 | 0.31**^*^** | (0.76) |  |
| 4.Excessive Consumption | 0.13 | -0.11 | -0.58**^**^** | (0.72) |
| Means | 4.67 | 4.58 | 4.49 | 4.40 |
| *SD* | (1.14) | (1.26) | (0.97) | (0.95) |

**Notes: Pearson bivariate correlations. Reliabilities are listed in parentheses on main diagonal. Means and *SD*s are listed in last two rows.** *^*^p* < 0.05, ^∗∗^*p <* 0.01 (two-tailed).

Table 3 | The regression analyses ruling out alternative explanations (study 3).

| **Constructs** | ***β*** | ***SE*** | ***t*** | ***p*** | ***LLCI*** | ***ULCI*** |
| --- | --- | --- | --- | --- | --- | --- |
| Positive affect | -0.04 | 0.01 | -0.36 | 0.72 | -0.0313 | 0.0216 |
| Negative affect | 0.07 | 0.02 | 0.04 | 0.10 | -0.0357 | 0.0372 |
| Materialism | 0.22 | 0.33 | 0.66 | 0.51 | -0.4401 | 0.8846 |
| Self-esteem | -0.60 | 0.30 | -1.96 | 0.06 | -1.2014 | 0.0008 |
| Trustworthiness | 0.12 | 0.09 | 1.78 | 0.07 | -0.0170 | 0.3417 |
| Product expertise | -0.04 | 0.07 | -0.52 | 0.61 | -0.1824 | 0.1061 |

****Related Links***

【It all comes back to you】：37seconds

<https://v.youku.com/v_show/id_XMTU0MzA5NzI4.html>

【Garbage classification】：33seconds

<http://baidu.iqiyi.com/watch/3463031702725026242.html?page=videoMultiNeed>

【news about red meat overconsumption】

<http://www.chinadaily.com.cn/hkedition/2018-03/23/content_35901803.htm>
